# Incisional Endometriosis: Diagnosed by Fine Needle Aspiration Cytology

**DOI:** 10.4103/0974-2727.72216

**Published:** 2010

**Authors:** P Veda, M Srinivasaiah

**Affiliations:** Department of Pathology, India; 1 General Surgery, ESI PGIMSR, Rajajinagar, Bangalore – 560 010, India

**Keywords:** Fine needle aspiration cytology, hysterotomy, incisional endometriosis

## Abstract

Incisional endometriosis (IE) is a rare entity reported in 0.03–1.08% of women following obstetric or gynecologic surgeries. Most cases reported in literature have appeared after cesarean sections and were often clinically mistaken for hernia, abscess, suture granuloma or lipoma. We hereby report a case of IE following a second trimester hysterotomy, which was diagnosed by fine needle aspiration cytology (FNAC). Our patient was 26 years old, presenting with a mass over anterior abdominal wall, associated with incapacitating pain during each menstrual cycle. FNAC showed epithelial cells, stromal cells and hemosiderin laden macrophages. Based on the typical history, clinical and cytological features, the diagnosis of IE was established. Wide surgical excision was done and the resulting rectus sheath defect was repaired. Patient was followed for 6 months during which time she was symptom free. This article also reviews the spectrum of cytological features and the rare possibility of malignant transformation that can occur in IE.

## INTRODUCTION

Incisional endometriosis (IE) is a rare entity reported in 0.03–1.08% of women who have undergone obstetric or gynecologic surgeries. Most cases reported in literature have appeared after cesarean sections. Clinically, IE is often mistaken for abscess, hernia, suture granuloma or lipoma. We hereby report a case of IE following a second trimester hysterotomy, which was diagnosed on fine needle aspiration cytology (FNAC). Review of literature shows that FNAC can be useful in the diagnosis of IE. However, the cytopathologist should be aware of the spectrum of changes that can occur in both endometrial glandular cells and stromal cells, while evaluating these cases.

## CASE REPORT

A 26-year-old lady presented with a mass over anterior abdominal wall, of 2 years duration. She complained of incapacitating pain in the swelling, which started a week before menstruation and subsided a week after her periods. She also reported a corresponding fluctuation in the size of the swelling during each cycle. The patient had two normal deliveries in the past. Her third pregnancy was terminated in the second trimester by a hysterotomy, 4 years earlier.

On examination, the swelling was located on the left side of the anterior abdominal wall, close to the previous pfannenstiel incisional scar. It measured 6×4 cm, was firm, had well-defined borders and was situated in subcutaneous plane. There was no clinical or sonological evidence of pelvic endometriosis.

**Figure 1:**
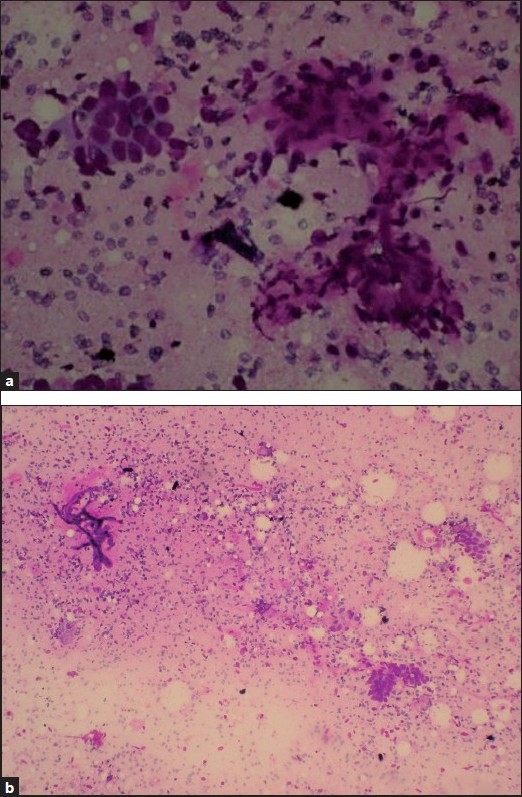
(a) FNAC smear from the abdominal wall swelling showing epithelial cells and stroma. Hemosiderin pigment seen in the background. MGG stain, ×45. (b) FNAC smear from the abdominal wall swelling showing epithelial cells and stroma. Capillaries seen within the stroma. Hemosiderin pigment seen in the background. MGG stain, ×45

FNAC showed sheets of epithelial cells and fibromyxoid stroma [Figure 1a and b]. The epithelial cells were uniform in size, with a moderate amount of cytoplasm. Nuclei were vesicular with inconspicuous nucleoli. The spindle-shaped stromal cells were loosely arranged in a meshwork of capillaries. Numerous pigment laden macrophages were also seen. Based on the typical history, clinical and cytological features, a diagnosis of IE was made.

Surgical excision was done, leaving a wide margin on all sides. Peroperatively, swelling was found attached to the anterior rectus sheath, part of which was also excised. The rectus sheath defect measuring 2×1 cm was repaired with number one proline. Recovery was uneventful.

**Figure 2:**
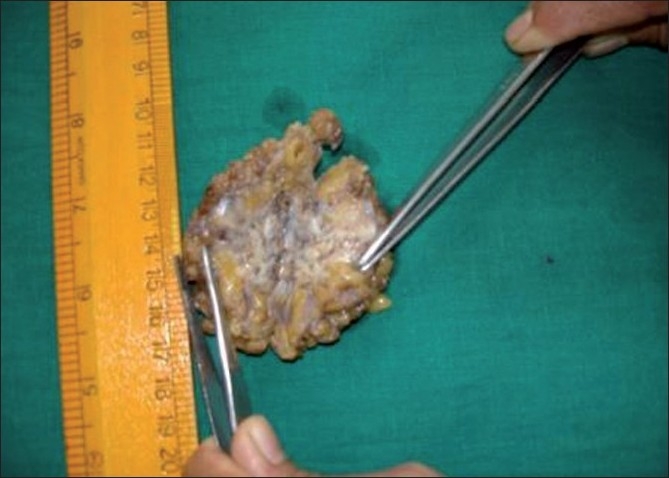
Excised specimen of the abdominal wall swelling. Cut section shows gray-white areas of IE surrounded by abdominal fat

**Figure 3:**
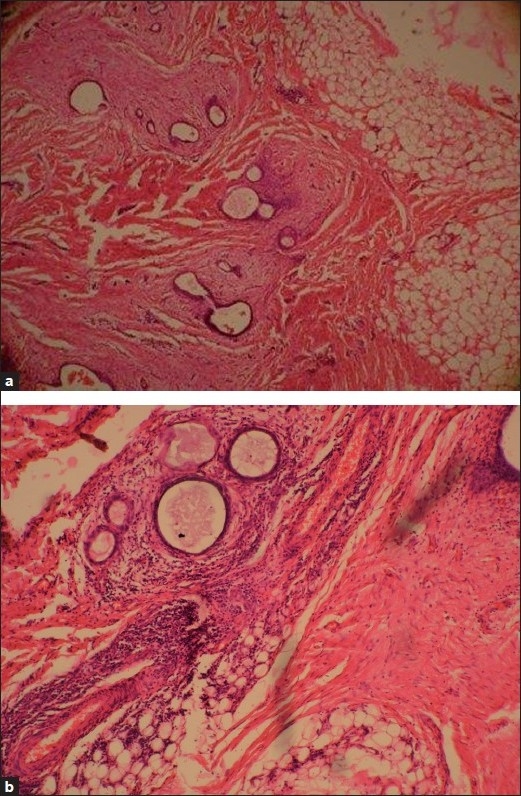
(a) Histological section showing cystically dilated endometrial glands surrounded by endometrial stroma. Adipose tissue of abdominal wall is also seen, H and E, ×4. (b) Histological section showing cystically dilated endometrial glands surrounded by endometrial stroma. Adipose tissue of abdominal wall is also seen, H and E, ×4

Grossly, the excised specimen was an irregular fatty mass, which on cut section showed a central fibrous area surrounded by multiple, tiny bluish spots [Figure 2]. Histopathology revealed cystic endometrial glands surrounded by endometrial stroma, embedded in the abdominal fat [Figure 3a and b]. The endometrial stroma showed focal myxoid changes. Hemosiderin pigment and areas of fibrosis were also seen. These features confirmed the diagnosis of IE. Patient was followed for 6 months after excision. She was completely relieved of her symptoms.

## DISCUSSION

Endometriosis refers to functional endometrial glands and stroma lying outside the uterine cavity. Endometriosis occurring in a surgical scar is called IE or scar endometriosis. An endometrioma refers to a circumscribed mass of ectopic endometrial tissue.[1] The presence of endometriosis in cesarean section scars have been documented in gynecologic literature since 1956. IE is underreported as it is often clinically mistaken for incisional hernia, suture granuloma, abscess, lipoma and other tumors.[2,3]

IE is reported to occur in 0.03–1.08% of women after obstetric or gynecologic surgeries, particularly after hysterotomy.[4] The incidence following cesarean section is reported to be 0.03–0.4%, and following hysterotomy, the incidence is 2%.[2,5] Chambers[5] has reported four cases of IE following hysterotomy, and has suggested that this higher incidence in hysterotomy is related to the regenerative capacity of the early pregnancy decidua, which gets implanted in the surgical wound. Experimental studies have shown that early pregnancy endometrium is easier to transplant than term pregnancy endometrium.[5] This could possibly explain the higher incidence of IE following hysterotomy, as compared to cesarean sections. Our patient had also undergone a second trimester hysterotomy.

IE has also been reported in scars resulting from hysterectomy, tubal ligation, ectopic pregnancy, salpingectomy, uterine suspension, inguinal herniorrhaphy, bartholin cyst excision, episiotomy, laprotomy, abdominoplasty, laproscopic trocar tract and needle tract following diagnostic amniocentesis.[6–8] Of the patients who develop IE, 25% have concomitant pelvic endometriosis.[9] It is observed that endometriomas can occur in the umbilicus even without antecedent surgery.[10] IE is a diagnostic pitfall and should be considered in the differential diagnosis of anterior abdominal wall masses.[9] Sometimes, the lesions may show blue black discoloration and ulcerate, leading to erroneous suspicion of malignancy.

The most common site of IE is near a pfannenstiel incision. This is possibly related to the wider dissection of the tissue planes when compared to vertical midline incision. Teng *et al*,[4] have reported 19 cases of IE in pfannenstiel incision, 18 of which were done for cesarean section and one for hysterotomy. Our case also developed IE in the pfannenstiel incisional scar done for hysterotomy.

Diagnosis of scar endometriosis is usually made on clinical grounds. In clinically doubtful cases, FNAC has been a valuable diagnostic tool. Cytology smears show sheets of epithelial cells, spindled stromal cells and a variable number of hemosiderin laden macrophages. The stromal cells are plump, spindled and arranged around a vascular meshwork. The presence of any two of the three components is required for the diagnosis of endometriosis.[11] The importance of FNAC lies in excluding other lesions like metastatic deposit, desmoid tumor, lipomas, sarcomas, hernias, cysts, myxoma, fat necrosis, hematoma or abscess.[11] Sometimes, the FNAC smears can be hemorrhagic showing only a few macrophages and inflammatory cells, in which case the diagnosis of IE can be missed. If only endometrial stroma is picked up, it could be mistaken for a stromal neoplasm.

The epithelial and stromal cells in IE can undergo a wide spectrum of changes which may pose diagnostic challenges. Epithelial cells may also undergo squamous, mucinous or tubal metaplasia. Nuclear atypia and cytoplasmic vacuolation has been reported in the glandular cells during secretory phase.[11] Stromal elements can develop decidual or myxoid changes.[11,12] Nogales *et al*,[12] have reported a case of cesarean scar endometriosis with massive decidualization in a 25-year-old patient, which presented as an extensively ulcerated lesion. This lesion mimicked malignancy microscopically due to myxoid change with alveolar pattern reminiscent of soft tissue sarcoma, signet ring like cells and pseudoinfiltration of the fascia. Malignant transformation is a rare but well-documented complication of IE. The malignancies reported include endometrioid carcinoma, clear cell carcinoma, sarcoma, mucinous and serous carcinoma.[13] If nuclear atypia is identified in IE, the mass should be excised and evaluated histologically to rule out malignancy.

Medical management with oral contraceptive pill, progestogens and gonadotropin releasing hormone analogues provide alleviation of symptoms, but recurrence is common after cessation of therapy. Wide surgical excision with at least 1 cm margin on all sides and patch grafting of the fascial defect, if necessary, is the treatment of choice.[4,8] It is believed that IE results from iatrogenic inoculation of the fascia or subcutaneous tissues with endometrial cells during invasive abdominopelvic procedures. Therefore, it is strongly recommended that before closure, the abdominal wound must be thoroughly cleaned and irrigated vigorously with saline.[4]
